# New Detector Sensitivity Calibration and the Calculation of the Interaction Force between Particles Using an Optical Tweezer

**DOI:** 10.3390/mi9090425

**Published:** 2018-08-24

**Authors:** Pavel Yale, Jean-Michel Edoukoua Konin, Michel Abaka Kouacou, Jérémie Thouakesseh Zoueu

**Affiliations:** Laboratoire d’Instrumentation, Image et Spectroscopie (L2IS), Institut National Polytechnique Houphouët-Boigny (INPHB), BP 1093 Yamoussoukro, Cote D'Ivoire; kedoukoua@yahoo.fr (J.-M.E.K.); abakaci@yahoo.fr (M.A.K.); jzoueu@yahoo.fr (J.T.Z.)

**Keywords:** detector sensitivity, calibration, silica bead, interaction force

## Abstract

We propose a new approach to calculate the sensitivity factor of the detector in optical tweezers. In this work, we used a charge-coupled device (CCD) camera and a quadrant photodiode (QPD) for the extraction of the various positions occupied by the trapped object (in this case, silica beads of different diameters). Image-J software and the Boltzmann statistical method were then used to estimate the sensitivity of the detector. Silica beads of diameter 0.8 µm, 2 µm, a system of 2 µm bead stuck to 4.5 µm one and another system of 2 µm beads stuck to 2 µm one, were studied. This work contributes significantly to making better calibration of the detector without taking into account the geometry of the object imprisoned in the optical trap. We further developed an approach to calculate the interaction force between two microbeads. This approach does not require any knowledge of solvent viscosity and works for all types of samples.

## 1. Introduction

Manipulation of biological specimens using microscopic light is a subject of increasing interest. This is due to its extensive applicability and relevance to fundamental research [1]. To study phase transitions in three dimensional systems and to measure directly the interaction between isolated pairs of colloidal microspheres, quantitative analysis of colloidal images has been used [2,3,4,5]. Also, for Colloidal Studies, Crocker and Grier [6] described a set of image processing algorithms for extracting quantitative data from digitized video microscope images of colloidal suspensions.

Recently, microfluidics [7], particle tracking [8], and holographic optical tweezers [9] have been combined to create a fully controlled and tunable environment to study passive diffusion [10].

Using optical tweezers, small cell or biological particles can be optically trapped and moved by exertion of Pico newton forces without causing any severe damage to the object of interest. The optical trapping mechanism is based on the momentum exchange between the trapped particle and the trapping beam using a scattering process. It is important to use a highly convergent laser beam focused into a sample chamber for the special case of a single-beam optical trap. When an oil-immersion objective is used for trapping in aqueous solutions, spherical aberration occurs because of the refractive index mismatch between oil and coverslip *n* = 1.55 and water *n* = 1.33 [11]. Practically in all applications of the laser trapping technique, it is important to have knowledge about the optical force used in trapping the particle. The optical trap therefore has to be calibrated for each type of trapped particle and laser power [12].This is achieved by relating the voltage output of the detector electronics to displacements and forces. The detector can then be calibrated accurately and the trap stiffness determined [13]. 

A widely used method to measure the detector calibration factor consists of moving a fixed bead over a known distance across the laser beam waist while recording the signals from the quadrant photodiode [14,15]. However, there are disadvantages related to using this method including (a) It is not possible to perform calibration with the same microbead and at the same position in the sample as the actual experiment, the latter being especially important when the focus gets distorted with increasing distance from the glass surface due to spherical aberration caused by the oil immersion objective; (b) It is critical, but difficult in practice, to position the fixed microbead correctly with respect to the beam laser in the three directions; and (c) The measured response is influenced when the trapped bead is near the cover slip [11,16]. One can also calculate the calibration factor based on the power spectral density (PSD), which is inferred from the Brownian motion of a trapped microbead [17,18].

Li and Arlt [19] studied a system of two stuck beads in the axial direction. Under these conditions, they used the diffusion coefficient of a trapped microbead $D_{0}=\frac{K_{B}T}{6\pi \gamma r}$ by supposing that the displacement from these two beads was independent of the viscosity coefficient. Additionally, for two stuck beads study in a side way, Koehler et al. [20] used an over relation to estimate the diffusion coefficient. Then, the approaches used in the case of the identical beads will present limits for beads of different sizes, which requires another approach. For a trapped silica bead of 2 µm diameter, Yale et al. [21] used the power spectra density method to estimate the quadrant photodiode (QPD) sensitivity factor at 24.16 mW and 25.26 mW laser power; the values obtained were, respectively, 0.1634 µm/V and 0.1746 µm/V. When fluid viscosity, bead diameter, and temperature are known, the PSD of Brownian motion is exactly predictable and can be used to calibrate the measured displacements of the bead in the trap [18]. According to Osterman [22], the Boltzmann method also provides information on the potential in the area far from the center of the trap where potential optics are non-harmonic. In this paper, we propose two approaches to determine easily detector calibration factors and to estimate the interaction force between particles. In Section 2.2.2, we used Boltzmann statistic to explain and calculate the induced forces and then estimate the interaction force between two stuck particles. In Section 2.2.3, we used also Boltzmann statistic to calculate trap stiffness for the data obtained, respectively, from the QPD and the charge-coupled device (CCD) camera. The report/ratio of these two trap stiffnesses made it possible then to estimate easily the detector calibration factors for each measurement.

## 2. Materials and Methods

### 2.1. Experimental Setup

**Figure 1:**
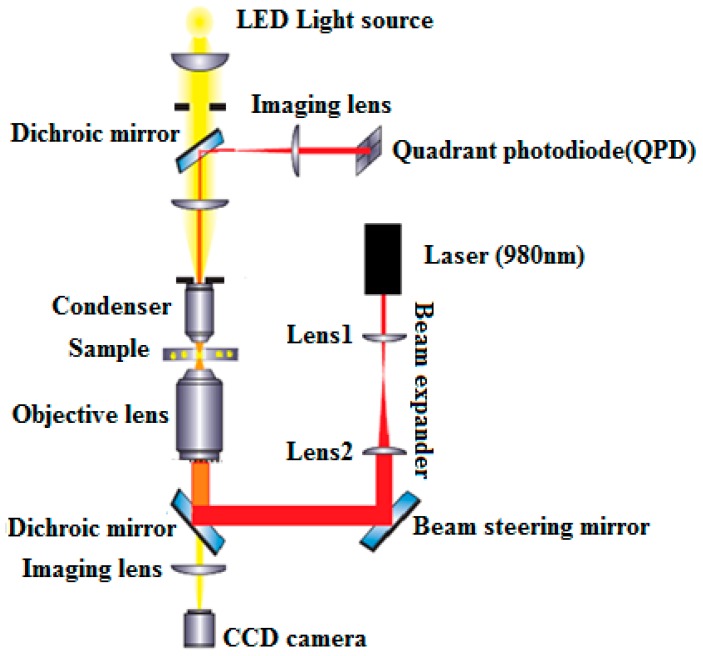
Setup scheme used for experiments.

In this section, we describe in detail the equipment and controls used for the experiment. Figure 1 shows the experimental setup for the optical tweezers used in this work. The optical tweezers setup consists of a Diode Laser (PL980P330J, Thorlabs, Newton, NJ, USA) at a wavelength of 980 nm with an output power of up to 330 mW. A large numerical aperture (NA 1.25) Nikon 100× oil immersion objective (MRP01902, Nikon, Tokyo, Japan) is used to focus a laser beam and form an optical trap. A white light-emitting diode (LED) source is mounted above the optical trap in order to illuminate a sample with light in the visible part of the electromagnetic spectrum. The forward-scattered light transmitted from the sample is collected by Nikon 10× air condenser. The sample is mounted on a 3-axis piezo translation stage (MAX301) with strain gauge feedback. The particles used in these experiments are silica beads with diameters of 0.8 µm, 2 µm, and 4.5 µm (Bangs Laboratories, Inc., Fishers, IN, USA). Samples for our experiment were prepared by diluting the silica beads in distilled water. The images of the beads were captured using a CCD camera and recorded onto a videotape. 

The video images were then downloaded onto a computer and digitized for image analysis. The individual frames of the recorded movies were analyzed by using the Image-J software (version 1.43, National Institutes of Health, Bethesda, MD, USA). The laser power measured after the objective used in our experiments ranged from 55.4 mW to 69.05 mW.

### 2.2. Methods

#### 2.2.1. Force Calibration

There are several independent methods that have been used to determine the trapping force based on the Brownian motion of a trapped particle [22]. However, in this work we used the Boltzmann statistics to obtain the trap stiffness. The optical potential reconstruction using Boltzmann statistics can be used to determine any continuous trapping landscape in the accessible region by thermal agitation [22]. In equilibrium, the probability density *p*(*x*) of the 1D particle position is given by

(1) $$p(x)dx=Ce^{\frac{-E(x)}{K_{B}T}}$$

in which *C* is a normalization constant and *E*(*x*) is the trap potential. The shape of *E*(*x*) can be obtained from the normalized histogram of the trapped bead positions as

(2) $$\begin{array}{l} E(x)=\frac{1}{2}k_{trap}{\left(x\right)}^{2}=-k_{B}T\ln(p(x))\\ \Rightarrow k_{trap}=\frac{-2k_{B}T}{x{\left(t\right)}^{2}}\ln(p(x)) \end{array}$$

In the case of the commonly used TEM_00_ Gaussian trapping beam, which results in a harmonic trapping potential, one can fit a parabola $y=ax^{2}+b$ to the data in the central region of the potential to extract the trap stiffness and check for possible deviations from the perfect harmonic shape. The stiffness coefficient *k_trap_* = 2*a*/*k_B_T* obtained in such manner is more accurate than Equation (2). Another advantage of such calibration is that it also gives information about the potential in the region away from the trap center where the optical potential is non-harmonic [22].

#### 2.2.2. How to Calculate the Contact Force?

**Figure 2:**
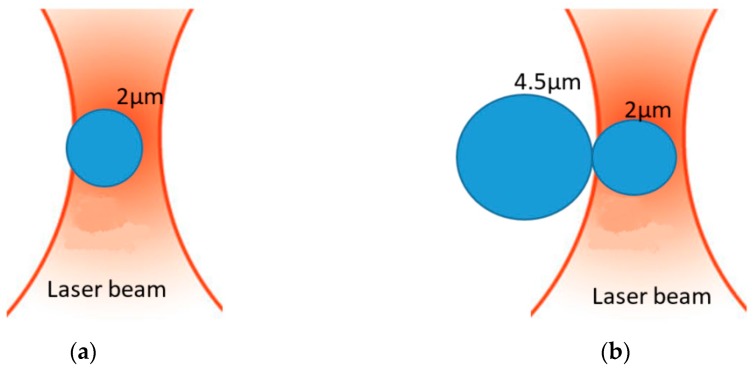
Illustration of different forces in competition: (**a**) 2 µm silica bead trapped and (**b**) system of a silica bead of 2 µm diameter stuck to one of 4.5 µm.

When the silica bead of 2 µm diameter is trapped, the silica bead of 4.5 µm diameter is brought closer towards the waist. The force of gradient thus tends to attract the silica bead of 4.5 µm in the focal point of the trap, and this bead thus will push back the bead of 2 µm (see Figure 2).

The sphere 1 (silica bead of diameter 2 µm), initially trapped, is found in a position of stable equilibrium. Since a stable equilibrium corresponds to a potential well, the method of Boltzmann's Statistics, which had been described in Section 2.2.1, can be used to explain and calculate the induced forces. At equilibrium, the potential well of sphere 1 is reconstructed with a potential written as $U_{1}=k_{1}{\left(\Delta r_{1}\right)}^{2}/2$. Interaction of the sphere 2 (silica bead of diameter 4.5 µm) with the sphere 1 will then disturb the initial equilibrium position of the sphere 1. In this new phase, the sphere 1 will have a new equilibrium position, with a potential well:

$U_{1}^{\prime }=\frac{k_{1}^{\prime }{\left(\Delta r_{1}^{\prime }\right)}^{2}}{2}\;\mathrm{with}\;k_{1},k_{1}^{\prime }and\;r_{1},r_{1}^{\prime }$ are, respectively, the stiffness constant of the optical trap and the position of the trapped sphere before and after the interaction.

Since it is the trapped sphere 1 that we can master, the energy communicated to sphere 1 by sphere 2 can be written according to the two potentials:

(3) $$U=U_{1}-U_{1}^{\prime }=k_{1}{\left(\Delta r_{1}\right)}^{2}/2-k_{1}^{\prime }{\left(\Delta r_{1}^{\prime }\right)}^{2}/2$$

As the force derives from the potential F=gradU, the interaction force can be written as follows:

(4) $$F_{int}=F_{1}-F_{1}^{\prime }$$

#### 2.2.3. Position Detection by Particle Tracking

**Figure 3:**
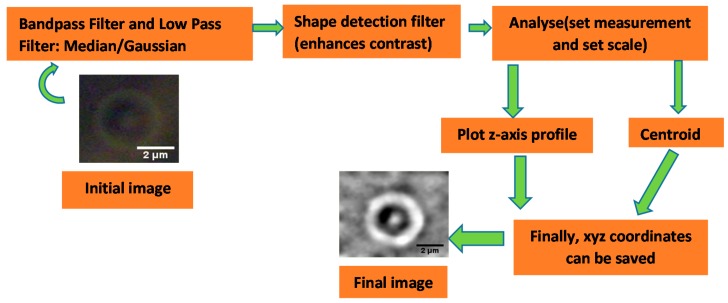
Image processing steps for particle center of mass detection for each recorded frame.

Particle tracking Image-J software has been used to reconstruct the *xyz*-path of trapped particle from a video recording. The result of a sequence of video frames is the *xyz*-path of each particle with pixel resolution (see Figure 3). The relationship between the distances in the image (measured in pixels) and the real distances at the trapping plane must be known before taking data. For the CCD camera used in this work, the linear relation between the distance and, in pixels, the real distance was found to be 0.0416 ± 0.0001 µm/pixel.

The QPD can detect the intensity variations due to the particle Brownian movement and provide an electronic measurement that is proportional to the x and y coordinates of the trapped bead. Data acquisition from the QPD is carried out with the standard software that is included with the electronic and nanopositioning parts, i.e., it includes the APT software package.

The desired calibration factor for displacement is

(5) $$\beta =\frac{k_{QPD}{}^{\left(N/\mathrm{Volt}\right)}}{k_{Camera}{}^{\left(N/\mu m\right)}};\beta (\mu m/v)$$

in which $k_{QPD}$ is the experimentally determined trap stiffness for the data obtained from the QPD, and $k_{Camera}$ is the trap stiffness for the data obtained from the CCD camera.

## 3. Results and Discussion

### 3.1. Detector Sensitivity Calculation

**Figure 4:**
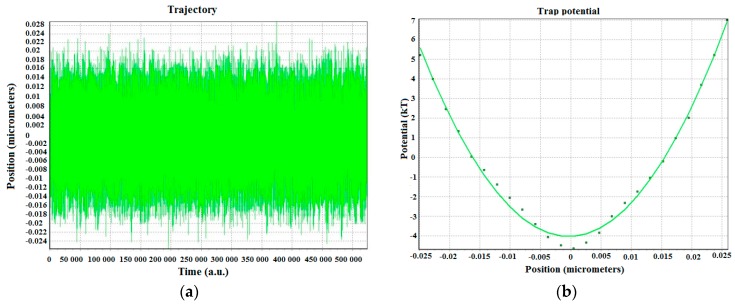
(**a**) Position of trapped silica bead and (**b**) typical potential well and fit of a trapped 2 µm diameter silica sphere at a depth of ≈5.25 µm from the glass surface with trapping laser power ≈69.05 mW.

Once the particle 2D-movement within the trap has been measured (with both QPD and video tracking), the optical trap can now be calibrated to obtain its transversal stiffness. The *X* and *Y* positions are those previously measured with both the QPD and the CCD camera (video tracking). This is done in order to compare their performance given the same experimental conditions. For example, 2 µm bead trapped at 5.25 µm height from the glass surface with 69.05 mW laser power at the sample. The QPD results describe almost an ideal Gaussian that has a huge amount of data (524,288 points) that can be collected in only 4 s. The video capture and tracking are much slower (20 fps). Hence, to compensate for the lower number of data points, the experiment has to be repeated several times in the same experimental conditions and the results averaged. The resulting typical potential well and fit are plotted in Figure 4.

**Figure 5:**
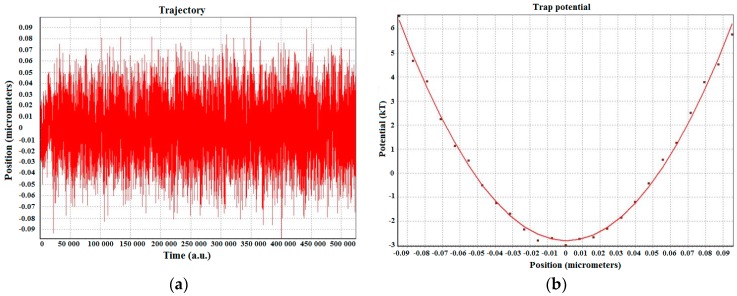
(**a**) Trajectory of the system of trapped silica beads and (**b**) the potential well and fit of a trapped 2 µm diameter silica bead attached to the silica bead of 4.5 µm diameter at a depth of ≈5.25 µm from the glass surface with trapping laser power ≈69.05 mW.

For the same experimental conditions as the 2 µm silica bead, a system of silica beads consisting of 2 µm diameter stuck to an over bead of 4.5 µm diameter were trapped so that data obtained with the QPD could be used to also measure more precisely the trap parameters (see Figure 5).

**Figure 6:**
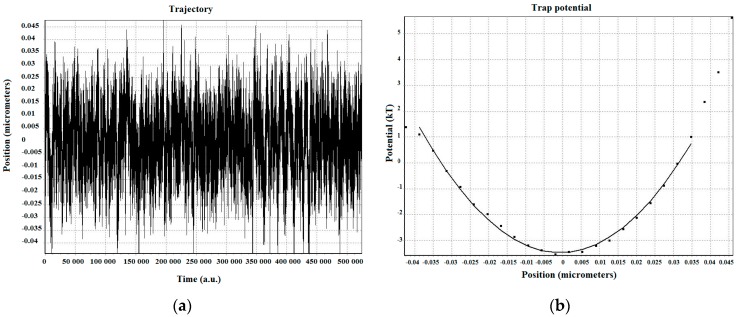
(**a**) Trajectory of trapped pairs of same diameter-sized silica beads and (**b**) the potential well and fit of the trapped 2 µm diameter silica bead attached to the silica bead of 2 µm diameter at a depth of ≈5.6 µm from the glass surface with trapping laser power ≈61.94 mW.

With the same experimental conditions as previously, two identical beads, both of diameter 2 µm, were studied. In Figure 6, we show by plotting the positions of the trapped pair of beads and the resulting potential well and fit.

The trap parameters for each laser power used are summarized in Table 1.

We observe that the trap stiffness obtained from harmonic fitting of *k* using the video tracking data represent 14% and 23% with respect to the final trap stiffness of 0.8 µm and 2 µm bead values obtained from QPD. Probably, this difference is introduced by the significant difference in the number of data points of each measurement. The same data recorded by the QPD were treated with the power spectral density method to calculate the sensitivity factor and trap stiffness. At 58.38 mW and 69.05 mW, the calculated stiffness was 0.1458 × 10^−4^ N/m and 0.47 × 10^−4^ N/m, respectively. External noise in the power spectrum measurements is clearly distinguishable; however, in this work, it was difficult to quantify and separate the noise from the real data. We can also affirm that this difference is introduced by the approach used in this work to calculate the QPD sensitivity factor, because it is based on Boltzmann statistics.

### 3.2. Determination of the Force Contact between Two Silica Beads

For the study of the two stuck beads, the data collected were also treated and used to estimate the different forces. Firstly, the force that the silica bead of 4.5 µm diameter exerts on the trapped silica bead of 2 µm diameter was obtained at each measurement. Secondly, the force with which the bead of 2 µm interacted with the trapped bead of diameter 2 µm was obtained for each measurement (see Table 2).

Here, it can be noticed that at each laser power, the Trap force values obtained for the trapped bead of 2 µm were within a factor of 2.5 of those obtained for the system of 2 µm bead stuck to 4.5 µm one. Also, we notice well that the trapping force obtained for the beads in interaction was almost unchanged over the power range examined, whereas it decreased with increasing bead diameter.

The value of (2.53 ± 0.02) pN is the minimal gradient force to maintain the silica bead of 2 µm diameter near to the waist, when it is interacting with the bead of 4.5 µm diameter (2 µm–4.5 µm). For the system of two beads having equal diameter (2 µm–2 µm), the minimal gradient force to maintain the silica bead of 2 µm diameter near to the waist is (1.33 ± 0.012) pN. The forces exerted by the bead of 2 µm on the imprisoned bead of 2 µm are higher than those exerted by the bead of 4.5 µm on the trapped bead of 2 µm. This difference between these values is due to the size of the beads interacting with that which is trapped. 

Better still, the Brownian movement of the bead of 2 µm is more intense than that of the bead of 4.5 µm. The difference in force is thus the gradient force available to attract the bead of 4.5 µm or 2 µm diameter towards the trap. By considering the trapped bead of 2 µm as being the system studied, the force that is available to attract the bead of 4.5 µm or 2 µm diameter is no different than the contact force between two beads in interaction.

**Figure 7:**
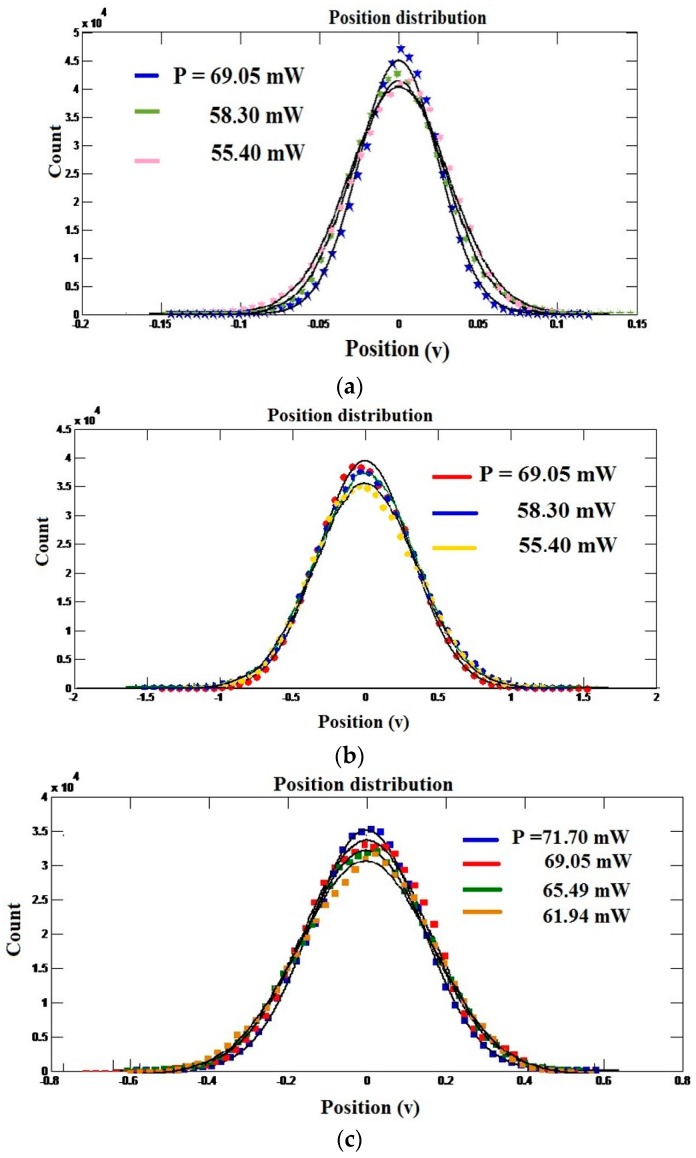
Statistical distributions for (**a**) microbead of 2 µm diameter, (**b**) two different microbeads stuck (2 µm–4.5 µm), and (**c**) pair microbeads of 2 µm diameter (2 µm–2 µm).

The equipartition method was further applied to these same preceding data. Figure 7 presents the statistical distributions of the trapped microbead position in the transverse direction. The experimental points were well adjusted with Gaussians functions. It can be noted that as the power of trapping is increased, the more the points are brought closer and the distributions are narrow. (See Figure 7a). That shows that the particle moves slightly, and thus the trap becomes stronger. 

In Figure 7b, we notice that for these three powers applied to trap the microbead of 2 µm, the statistical distributions practically coincide, revealing very weak variation in the trap stiffness. In Figure 7c, only statistical distribution results from 71.70 mW, which is different from the others. This increase in trapping force can be explained by the fact that the two microbeads are firmly dependent and thus produce an overall movement. This method further confirms what was found previously with the Boltzmann statistics method. 

## 4. Conclusions

The proposed approach, which simultaneously uses the data obtained with the camera and the quadrant photodiode, makes it possible to easily estimate with precision the conversion factor of the QPD at each measurement. Moreover, this approach also calibrates the detector for any kind of trapped particles. The difference between this and other methods is that the optical potential reconstruction based on the histogram is also applicable in non-harmonic optical potentials, because we can measure any kind of optical landscape that is accessible by thermal agitation. Another advantage is that the only experimental parameter that must be known is temperature; there is no need to know other sample parameters like medium viscosity, distance to the cover-slip, or particle size, because the thermal agitation of the trapped bead can directly be measured. The approach used to calculate the interaction force between microbeads could thus be a genuine tool to estimate the forces exerted on the biological cells at the time of their deformation. This proposed method could be also applicable to an optical trap integrated in a microfluidic chip.

## Figures and Tables

**Table 1:** Summary of trap parameters measured in x direction.

Bead Diameter	Laser Power	Depth (µm)	Trap Stiffness *k_QPD_* (N/V)	Trap Stiffness *k_Camera_* (N/m)	Sensibility Factor *β* (µm/V)	Final-Trap Stiffness *k* (N/m)
0.8 µm	55.40	2.17	2.60 × 10^−12^	2.12 × 10^−4^	0.0234	(4.00 ± 0.02) × 10^−3^
	69.05	6.58	2.50 × 10^−12^	5.80 × 10^−4^	0.0232	(4.14 ± 0.02) × 10^−3^
2 µm	69.05	5.25	3.38 × 10^−12^	4.47 × 10^−5^	0.1322	(1.94 ± 0.02) × 10^−3^
	58.38	5.21	3.30 × 10^−12^	4.59 × 10^−5^	0.1386	(1.74 ± 0.02) × 10^−4^

**Table 2:** Summary of trapping force and contact force measured in x direction for two silica beads in interaction.

Bead Diameter	Power (mW)	Trap Stiffness (N/m)	Trap Force (pN)	Contact Force (pN)
2 µm	55.40	(1.23 ± 0.01) × 10^−4^	6.330 ± 0.030	-
	58.38	(1.74 ± 0.02) × 10^−4^	6.977 ± 0.039	-
	69.05	(1.94 ± 0.02) × 10^−4^	7.180 ± 0.036	-
2 µm–4.5 µm	55.40	(5.08 ± 0.01) × 10^−5^	2.529 ± 0.022	3.801 ± 0.052
	58.38	(5.63 ± 0.01) × 10^−5^	2.533 ± 0.024	4.444 ± 0.063
	69.05	(5.77 ± 0.02) × 10^−5^	2.540 ± 0.025	4.640 ± 0.061
2 µm–2 µm	61.94	(2.75 ± 0.01) × 10^−5^	1.340 ± 0.020	4.540 ± 0.060
	65.49	(3.33 ± 0.01) × 10^−5^	1.336 ± 0.021	4.560 ± 0.058
	69.05	(3.85 ± 0.03) × 10^−5^	1.341 ± 0.018	5.840 ± 0.061
